# A Highly Controllable Electrochemical Anodization Process to Fabricate Porous Anodic Aluminum Oxide Membranes

**DOI:** 10.1186/s11671-015-1202-y

**Published:** 2015-12-26

**Authors:** Yuanjing Lin, Qingfeng Lin, Xue Liu, Yuan Gao, Jin He, Wenli Wang, Zhiyong Fan

**Affiliations:** Department of Electronic and Computer Engineering, The Hong Kong University of Science and Technology, Clear Water Bay, Kowloon, Hong Kong SAR, China; Peking University Shenzhen SOC Key Laboratory, PKU-HKUST Shenzhen-Hong Kong Institution, Shenzhen, 518051 China; College of Textile and Clothing Engineering, Soochow University, Suzhou, 215021 China; National Engineering Laboratory for Modern Silk, Suzhou, 215123 China

**Keywords:** Anodic aluminum oxide, Nanoporous structure, Integrated charge density, Controllable electrochemical anodization

## Abstract

Due to the broad applications of porous alumina nanostructures, research on fabrication of anodized aluminum oxide (AAO) with nanoporous structure has triggered enormous attention. While fabrication of highly ordered nanoporous AAO with tunable geometric features has been widely reported, it is known that its growth rate can be easily affected by the fluctuation of process conditions such as acid concentration and temperature during electrochemical anodization process. To fabricate AAO with various geometric parameters, particularly, to realize precise control over pore depth for scientific research and commercial applications, a controllable fabrication process is essential. In this work, we revealed a linear correlation between the integrated electric charge flow throughout the circuit in the stable anodization process and the growth thickness of AAO membranes. With this understanding, we developed a facile approach to precisely control the growth process of the membranes. It was found that this approach is applicable in a large voltage range, and it may be extended to anodization of other metal materials such as Ti as well.

**Electronic supplementary material:**

The online version of this article (doi:10.1186/s11671-015-1202-y) contains supplementary material, which is available to authorized users.

## Background

Metal anodization has been broadly used in industry as a surface treatment technique to render materials with resistance against uncontrolled oxidation, abrasion, and corrosion. Although this technique has been developed for a long time, it was until 1990s that researchers discovered that highly ordered nanoporous structures can be achieved by properly tuning anodization conditions including electrolyte composition and concentration, temperature, as well as anodization voltage [1]. Among all valve metals that can be anodized, aluminum (Al) and titanium (Ti), particularly Al, can be anodized into nanoporous structures with well-controlled diameter, pitch, and depth. Membranes consist of these nanostructures, i.e., anodic titanium oxide (ATO) and anodic aluminum oxide (AAO), have wide nanoengineering applications that have attracted enormous attention. For example, AAO membranes have been used as templates to directly assemble semiconductor nanowires and nanorods for photodetection [2] and solar energy conversion [3–5]. A large internal surface area of these oxide nanostructures can also be harnessed to build high-performance energy storage devices such as Li-ion batteries [6] and supercapacitors [7, 8]. Meanwhile, it is worth pointing out that AAO structures can be engineered into a number of variants via proper combination of wet chemical etching and anodization processes. These variants include nanowells [9], inverted nanocones [10, 11], nanobowls [12], nanospikes [13–15], and the integrated nanopillar-nanowell structures [16]. These structures have been used as scaffold of energy harvesting and storage devices in our past works.

There are certainly a number of advantages of using porous anodic nanostructures, particularly AAO membranes for nanoengineering applications, such as large surface area, high regularity and scalable low-cost production [17]. In many applications, precise control of nanostructure shape and geometry is critical. It is known that the geometric features of porous AAO membranes are widely tunable via electrochemical anodization process [10]. Factors such as applied voltage, acid type, and concentration contribute to the formation of the porous nanostructures with various barrier layer thicknesses (*D*_*b*_), interpore distances (*D*_int_), and periodicity [18–20]. Meanwhile, it is noteworthy that the growth rate of porous AAO nanostructures is not constant during the entire growth process, even when the growth starts with a fixed voltage and given electrolyte. This can be attributed to two competing factors. On one hand, AAO growth rate is sensitive to electrolyte temperature. In general, higher temperature expedites growth and lower temperature slows down growth. Therefore, environmental temperature fluctuation leads to the variation of growth rate [19]. Besides, anodization current flow through electrolyte causes temperature increase. This should be also incorporated into consideration as well, particularly for high voltage/high-current anodization. On the other hand, during the anodization process, electrolyte composition is being gradually changed. Specifically, Al cations will be injected into electrolyte and hydrogen evolution at the cathode that reduces proton concentration in electrolyte [21]. The composition and concentration change of electrolyte inevitably affects anodization rate. All these complicated factors pose a challenge to precisely control anodization process, especially when there is a stringent requirement on final membrane thickness. In this work, we revisited the electrochemical reactions during Al anodization. Then a generic electric charge integral approach was developed to monitor the growth pore depth (*D*_*p*_) of AAO in real time. This approach is based on the discovered linear correlation between measured total electric charge flow through the circuit during stable anodization process and the molar amount of anodic alumina grown in the process. It was found that this linear correlation is rather insensitive to anodization voltage and composition of the electrolyte. This suggests that for a given anodization process, AAO pore depth can be predicted in real time regardless of the temperature variation and electrolyte concentration change. Therefore, a program was coded to visualize the AAO growth process. In this case, not only the AAO growth pore depth can be monitored, but also a target pore depth can be set and the anodization process can be terminated when the projected pore depth reaches the set point. Overall, the revealed correlation between the quantities of electric charge and anodic material is of scientific and practical importance, and it facilitates precise control of AAO anodization in a wide voltage range for a broad applications.

## Methods

**Fig. 1:**
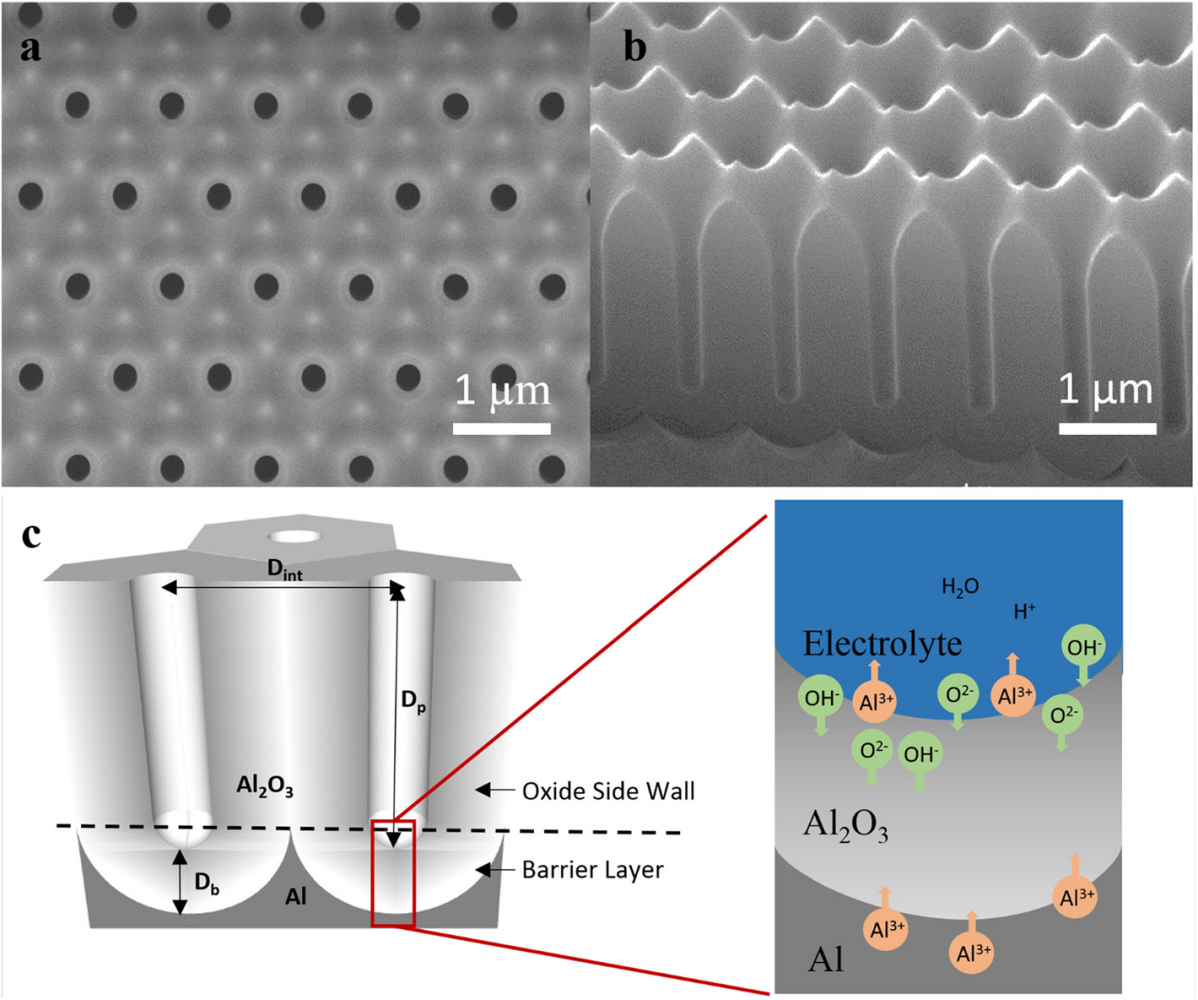
SEM images of imprinted AAO and schematic of AAO structure and formation mechanism. **a** Top-view SEM image of imprint AAO with *D*_int_ of 1 μm. **b** Cross-sectional SEM image of imprint AAO with *D*_int_ of 1 μm. **c** Schematic of AAO nanoporous structure and major features of AAO formation

The fabrication of porous AAO membranes with nanostructures mainly follows a two-step electrochemical anodization process [22] after proper pretreatments [23, 24]. The aluminum foils are initially covered by natural oxide layers and surface roughness caused by thermal and mechanical processes [25]. To minimize the impact of these defects on the fabrication of highly ordered porous AAO membranes, pretreatments such as pressing and electropolishing play an essential role in removing particles and smoothing the surface of aluminums. Normally, the two-step anodization of aluminum leads to formation of AAO nanopores with short-range hexagonal ordering [26]. Typically, *D*_int_ is simply proportional to the applied voltage (*U*) with a linear constant of 2.5 nm/V, namely ${D}_{\mathrm{int}}(nm)=2.5\left(\frac{nm}{V}\right)\times U(V)$ [18]. Meanwhile the depth of the nanopores and their diameter can be controlled by anodization time and the subsequent wet chemical etching. In some applications, highly ordered structures are required. For example in nanophotonic applications, both ordering and periodicity may affect light-structure interaction [9]. Moreover, when utilizing nanopores to directly integrate semiconducting nanowires for nanoelectronic applications, each nanowires needs to be individually addressable which also requires perfect ordering. In the past, we have intensively explored fabrication of AAO membranes with perfect hexagonal and square orderings with an area up to tens of centimeter square, and the periodicity ranging from 500 nm to 3 μm [9, 10, 13, 14, 27]. In order to achieve long-range regular structures, hexagonally or squarely ordered nanohole arrays are produced on electrochemically polished aluminum surface with nanoimprint, using a silicon stamp mold with ordered short pillars on the surface. Then the aluminum sample undergoes anodization process yielding a perfectly ordered AAO nanopore array on the surface. Note that it is important to satisfy a matching condition between the periodicity of the nanoimprinted nanoholes and the anodization voltage, governed by the linear constant of 2.5 nm/V. Figure 1a, b shows the scanning electron microscopic (SEM) images of an as-fabricated AAO membrane demonstrating ideally regular configuration of the straight and parallel holes. In this case, the Al foil was anodized with an applied voltage of 400 V in 230 ml 1:1(*v*/*v*) mixture of 4 wt.% citric acid (C_6_H_8_O_7_) and ethylene glycol (EG) with an extra 15 ml 0.1 wt.% phosphoric acid (H_3_PO_4_) at a temperature of 10 °C for 30 min.

In order to have a more precise control of the anodization process in this work, the voltage and current of the anodization process are monitored and recorded by Keithley 2410 SourceMeter, controlled by a home-built computer program based on LabView. The voltage was increased in a linear manner from zero to the target value with a ramping rate of 10 V/min, so that the current will not rise too fast to avoid overheating of the electrolyte and burning aluminum samples. Meanwhile, we have also discovered that a ramping process which lasted too long will result in forming a fairly smooth anodic oxide film without nanoporous structure. The unnecessarily slow voltage ramping cannot provide high enough electric field for expelling Al^3+^ into electrolyte, and the Al^3+^ is retained in oxide bulk. Thus, the oxidization is fairly stable without variation in electric field distribution on the oxide layer and therefore hinders the formation of hemispherical oxide layer and nanoporous structure [25]. Once the ramping process is done, the program maintains target voltage while it is observed that the anodization current falls naturally down to a stable level, which is an indication of inception of stable anodization [28]. In our experiment, a two-step anodization approach is adopted. In the first step, an initial AAO layer was formed after anodization in electrolyte for over 12 h. The first layer of AAO was then removed in a mixture of 6 wt.% phosphoric acid (H_3_PO_4_) and 1.8 wt.% chromic acid (H_2_CrO_4_) at 98 °C for 30 min, leaving a highly periodic nanoconcave structure on the surface of the aluminum substrate, which forms the initiation sites for the formation of pores in the second anodization step. Interestingly, this nanoconcave structure can be used to enhance light absorption and power conversion efficiency of thin-film solar cells which was reported by us previously [29]. Furthermore, AAO membrane with highly ordered porous nanostructure is produced in the same electrolyte during second anodization step.It is well known that an AAO membrane contains two parts of oxide layers, namely barrier layer and the oxide side wall, as shown in Fig. 1c. AAO is mainly formed at the AAO/Al interface when OH^−^ and O^2−^ diffuse through the barrier layer driven by the electric field [25]. The thickness of the barrier layer remains constant for a particular anodization voltage during the stable anodization process, while the length/height of the oxide side wall continues to grow. In the first step anodization, the nanoimprinted nanoholes on the aluminum surface are the centers for concentrated electric field. During the initial voltage ramping process, an oxide barrier layer with hemispherical shape is formed due to radial alignment of electric field line vectors. When the oxide barrier layer is thin, electric field is strong; thus, the field driven diffusion of OH^−^ and O^2−^ to AAO/Al interface is fast leading to fast oxidation of Al and increase of barrier layer thickness. At the AAO/electrolyte interface, field-assisted etching of AAO and thinning of the barrier layer occur since dissolution of Al_2_O_3_ in pH <5 electrolyte [28]. It is obvious that the above barrier layer growth and etching processes are the two competing processes. The etching rate is not only a function of local electric field intensity, local pH value, but also highly depends on temperature. In practice, the temperature is kept at 5~10 °C to slow down the barrier layer dissolution. Generally, the barrier layer growth rate is largely determined by electric field intensity. In the beginning of the barrier layer growth, small oxide thickness leads to high electric field thus, the overall barrier layer thickness increases over the time. However, after a certain period of time, the electric field intensity drops to a level so that a balanced barrier oxide layer dissolution and growth is achieved. Thereafter, the barrier layer thickness remains constant. As a natural outcome of this dynamic process, the AAO/Al interface moves deeper and deeper into Al bulk, leading to a sustainable growth of pore depth. This formation mechanism explains tubular structure of each AAO nanochannel, and the side-wall thickness is roughly twice of that of the barrier layer, since half of the side wall comes from the barrier layer. It is interesting to notice that the hexagonal nanopore arrangement is typically observed even without assistance of nanoimprint, though in a short range. This can be explained by the volumetric expansion of nanopores during formation of oxide which induces stress between neighboring pores [30]. In order to achieve minimal system energy, a close-pack hexagonal ordering is naturally formed.

## Results and Discussion

**Fig. 2:**
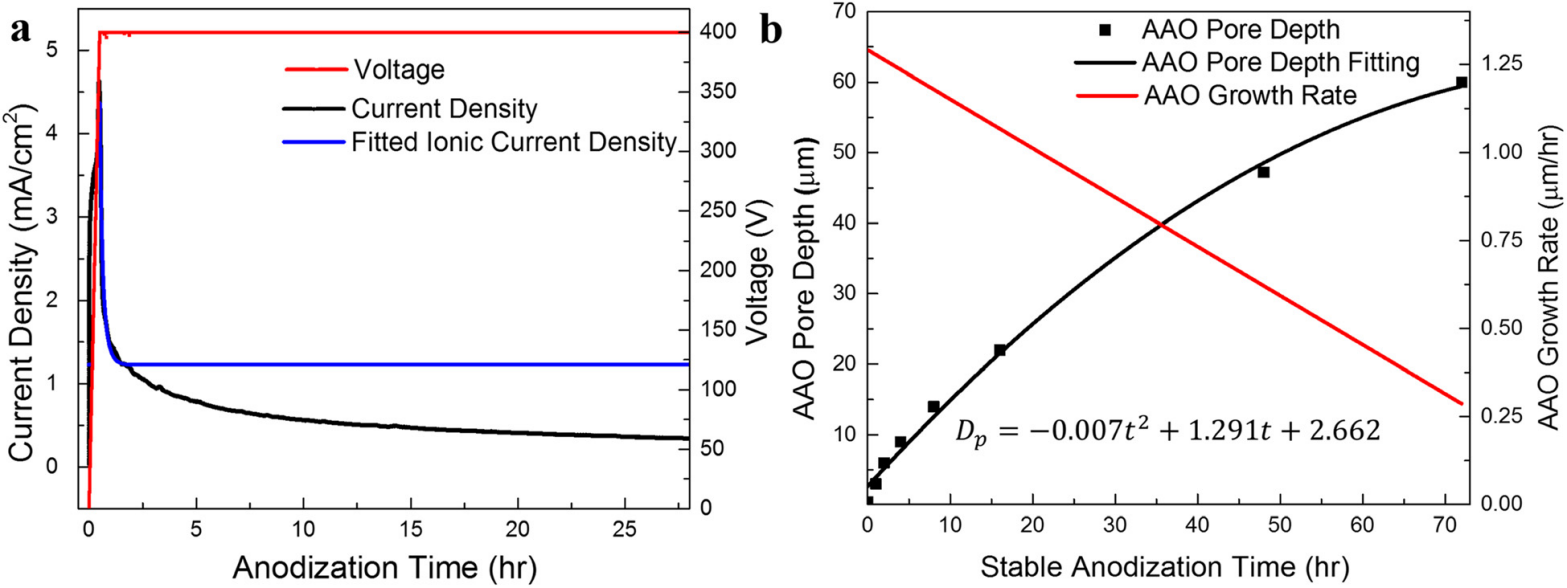
Temporal response of anodization current and voltage and AAO growth rate. **a** Current density and voltage curve versus time. Ionic current is the dominated contributor to the current flow. **b** AAO pore depth and growth rate over stable anodization time

Figure 2a shows measured current density-time (J-t) and voltage-time (V-t) curves recorded during a 400 V anodization process. According to the AAO growth mechanism discussed above, ionic current is the dominant contributor to the observed current flow while electronic current can be ignored since AAO itself is an insulator [31]. The transportation of anions (O^2−^, OH^−^) and cations (Al^3+^) contributes to most of the ionic current. It has been reported that the ionic current density is related to electric field strength (*E*) with an exponential function [21]:

1 $$ {J}_{\mathrm{ion}}= An{e}^{BE}= An{e}^{\frac{BU}{D_b}} $$

where both *A* and *B* are constants related to electrolyte temperature, *n* is the surface density of mobile ions, *U* is the potential drop across the barrier layer, and *D*_*b*_ is the thickness of the barrier layer. At the moment (*t*_0_) when an equilibrium state of dissolution and oxidization is established, the typical barrier thickness (*D*_*b*0_) is proportional to applied voltage with a linear constant (*λ*_*b*_) approximately ranges from 1.0 to 1.4 nm/V depends on electrolyte components and temperature [21], namely, ${D}_{b0}\ (nm)={\uplambda}_b\left(\frac{nm}{V}\right)\times V(V)$. The growth rate of the barrier layer can be defined as [32]:

2 $$ \frac{\mathrm{d}{D}_b}{\mathrm{d}\mathrm{t}}=\left\{\begin{array}{c}\hfill {G}_0-\frac{D_b}{\tau}\left({D}_b<{D}_{b0}\right)\hfill \\ {}\hfill \kern0.5em 0\kern2.5em \left({D}_b={D}_{b0}\right)\hfill \end{array}\right. $$

Where *G*_0_ is the initial growth rate at the start of a stable applied anodization voltage, *τ* is a time constant. After integral, formula (2) can be rewritten as:

3 $$ \begin{array}{l}{D}_b=\left\{\begin{array}{c}\hfill {G}_0\tau \left(1-{e}^{-\frac{t}{\tau }}\right)\kern0.72em \left(\mathrm{t}<{t}_0\right)\hfill \\ {}\hfill {D_b}_0\kern3.12em \left(\mathrm{t}\ge {t}_0\right)\hfill \end{array}\right.\\ {}\kern1.56em \end{array} $$

Therefore, after combining Eqs. 1 and 3, the ionic current density can be expressed as:

4 $$ {J}_{\mathrm{ion}}=\left\{\begin{array}{c}\hfill An{e}^{\frac{BU}{G_0\tau\ \left(1-{e}^{-\frac{t}{\tau }}\right)}}\left(\mathrm{t}<{t}_0\right)\hfill \\ {}\hfill An{e}^{\frac{BU}{D_{b0}}}\kern2.75em \left(\mathrm{t}\ge {t}_0\right)\hfill \end{array}\right. $$

The current density curve can be mathematically fitted with the Eq. 4 as shown in Fig. 2a, and the fitted expression is:

5 $$ {J}_{\mathrm{ion}}=\left\{\begin{array}{c}\hfill 1.47\times {10^{-4}}^{\frac{9.03}{\ \left(1-{e}^{-4.18t}\right)}}\left(\mathrm{t}<{t}_0\right)\hfill \\ {}\hfill 1.22\kern3.12em \left(\mathrm{t}\ge {t}_0\right)\ \hfill \end{array}\right. $$

The fitting proved that the ionic current is the main contributor to the growth of AAO, and the ionic current should remain unchanged during stable anodization process. However, we notice that the measured current density has a decaying nature in stable anodization process. This can be ascribed to the gradually reduced concentration of H^+^ in electrolyte and as a result, the dissolution rate together with oxidation rate is continuously slowed down.

**Fig. 3:**
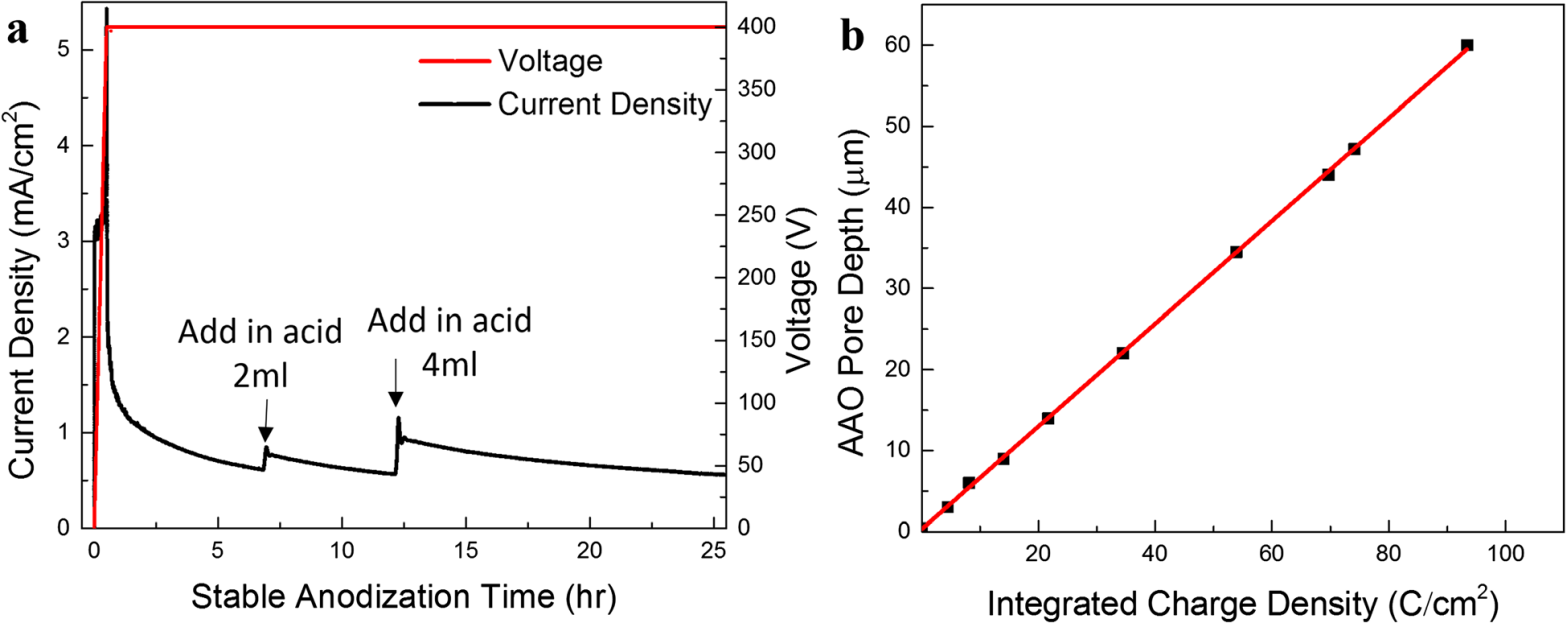
Temporal response of anodization current and voltage response with perturbation on anodization conditions. **a** Current density and voltage temporal response with adding acid twice during the process. **b** Linear fitting of AAO pore depth dependence on integral of stable anodization charge density

To investigate the growth rate of AAO pore depth, seven AAO membranes were grown with different anodization time under an applied voltage of 400 V and a temperature of 10 °C. Figure 2b shows AAO pore depth, which is roughly AAO thickness, versus anodization time with polynomial fitting. It is obvious that the growth rate of AAO is not constant even without fluctuation in environmental conditions. As a result, a declining growth rate can be derived and plotted, as shown in Fig. 2b. One of the effective ways to recover the growth rate after a certain thickness of AAO has grown is to replenish acid during the reaction to compensate proton loss. As shown in Fig. 3a, immediate current rise can be recorded upon admittance of 2 and 4 ml 0.1 wt.% H_3_PO_4_ after 7 and 12.5 h, respectively, which indicates an accelerated oxidation rate of AAO growth. With an electric field established in the electrolyte, additional protons from the replenished acid quickly migrate to cathode leading to moving extra amount of electrons from Al anode through external circuit, which is indicated by the fast current rise. Once the new charge distribution is established, the current declines. Overall, the amount of protons at the proximity of cathode and oxygen containing anions (PO_4_^3−^) at the AAO/electrolyte interface increased and accelerated the anodization reaction. This explains the observation of a current spike followed by a step increase of anodization current. It is obvious that fluctuation of anodization conditions brings in difficulties in predicting the real-time AAO pore depth during the anodization process. Considering the fact that the measured electric current in external circuit is equal to the ionic current in magnitude, the electric current can be used to indicate the growth rate of AAO pore depth. Since the integral of grow rate over time leads to the pore depth, the integral of current density in principle can also be used to indicate AAO pore depth. As a matter of fact, the relationship between AAO pore depth and the integrated charge density is shown in Fig. 3b, which clearly suggests a linear relationship between the pore depth and charge density. Therefore, this linear relationship can be utilized to monitor AAO pore depth in real time.

The validity of this linear correlation was further verified with anodization under applied voltage ranging from 20 to 600 V in different electrolytes (see Additional file 1: Figure S1). The slope of the linear fitting was defined as growth constant (*G*), which has a physical meaning of increment of AAO membrane volume per unit charge and can be theoretically estimated. In experiments, we have observed small intercepts on the AAO pore depth (*Y*) axis with value ranging from 30~500 nm. This is caused by initial unstable anodization of Al before the constant thickness of the barrier layer is established. Specifically, the volume of AAO (*V*_*A*_) can be expressed as:

6 $$ {V}_A=\frac{n\times M}{\rho } $$

where *ρ* is the mass density of Al_2_O_3_, *n* is the molar amount of Al_2_O_3_ produced in oxidation, and *M* is the molar mass. In an ideal case, all the charges come from the oxidation of Al and are irrelevant to the dissolution of Al_2_O_3_ and thus, the charge quantity (*q*) has a linear relationship with *n* as:

7 $$ q=6\mathrm{e}{N}_A\times n $$

where *e* is the elementary charge and *N*_*A*_ is the Avogadro constant. Therefore, combining Eqs. 6 and 7, the theoretical growth constant can be calculated by using Eq. 8:

8 $$ G=\frac{{\mathrm{dV}}_A}{\mathrm{dq}}=\frac{M}{6e{N}_A\times \rho } $$

**Fig. 4:**
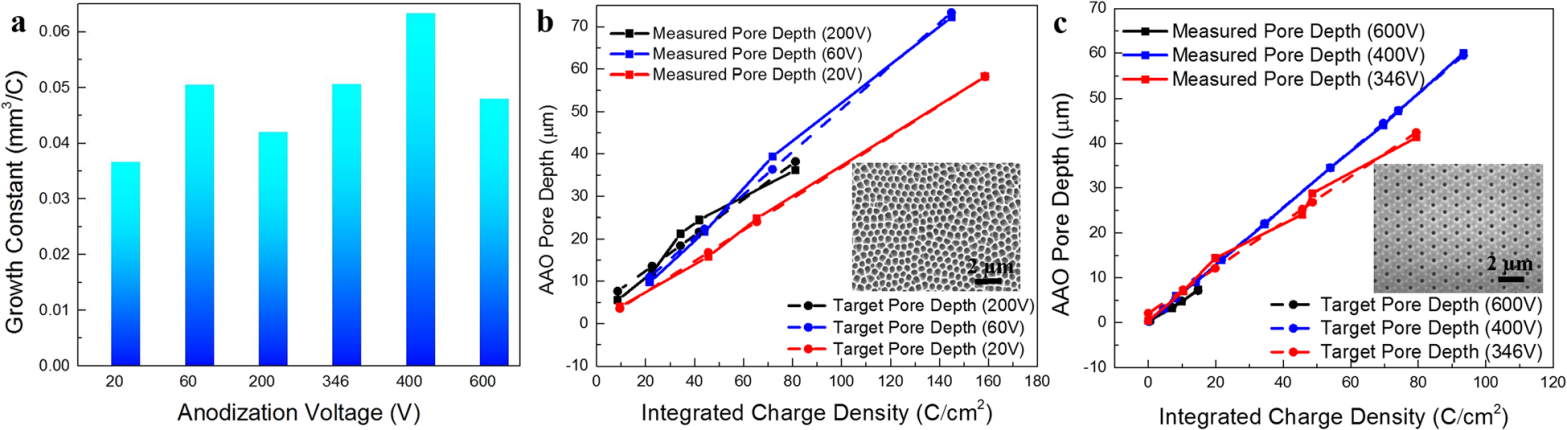
Growth constant and controlled AAO growth. **a** The measured growth constant of AAO membrane under different applied voltages. **b** Measured pore depth of non-imprinted AAO is consistent with target values. **c** Measured pore depth of imprinted AAO is consistent with target values

It can be seen from Eq. 8 that in theory, *G* is a constant independent of anodization condition. Specifically, the growth constant *G* (mm^3^/C) is theoretically independent of variations on factors such as acid concentration, temperature, and AAO pore diameter. While the growth rate (μm/h) of AAO membrane can be accelerated by higher acid concentration and electrolyte temperature, the integral of charge quantity from Al oxidation is independent of oxidation rate. Therefore, the linear correlation will not be affected even when there is a fluctuation in acid concentration and temperature. However, in experiments, we have discovered that excessive temperature leads to high current and burning of AAO due to uncontrollable fast anodization. The integral of charge density is not affected by variation of pore diameter and the porosity of AAO membrane, which indicates the linear correlation could be applied in fabrication of both non-imprint and imprint AAO membranes. However, the measured *G* obtained from Additional file 1: Figure S1a and b shows variation for different anodization voltage, as it can be clearly seen from Fig. 4a. We have attributed the variation of growth constants under different anodization voltages to mass density (*ρ*) deviation of AAO membrane obtained with different voltages. It is known that incorporation of anions and water molecules from the electrolyte into the oxide layer occurs during anodization process which changes mass density of AAO [33, 34]. It is reported that minor constituents such as anions from the electrolyte can be as much as 20 % of the AAO wall structure in volume depending upon the electrolytes and the growth conditions [35] and the amount of incorporation depends on electric field and acid concentration, etc.

With the achieved understanding on growth constant, the control of AAO membrane thickness can be realized with a programmable source-meter unit. Here, we have performed a set of AAO anodization with various target pore depths. Utilizing the measured growth constant *G*, pore depth is monitored by a computer connected with Keithley source-meter unit in real time and anodization processes were terminated automatically when reaching target AAO thickness. Figure 4b, c indicates that in a large voltage range and regardless of pore diameters, with this charge integration approach, the AAO pore depths can be precisely controlled with high consistency with the actual thickness measured with SEM. The marginal deviations may possibly come from: (1) inaccuracy in measuring the AAO pore depth with SEM, mainly from viewing angle deviation for cross-section measurement; and (2) deviation in fitting method. All the curves were fitted in concise models such as linear and exponential fitting so as to indicate the primary trend and thus, the measured growth rates derived from the fitting result could have a reasonable deviation.

## Conclusions

In this work, a linear correlation between AAO pore depth and integrated charge density during electrochemical anodization process was discovered and utilized to implement precise control of AAO growth process. This method proved to be repeatable and robust regardless of anodization voltage, temperature, and electrolyte composition and concentration variation. Thus, a home-built automatic AAO fabrication setup combining a source-meter unit and software was developed to realize real-time monitoring and automatic anodization termination when reaching a target pore depth. The good reliability in control of the as-fabricate AAO membrane thickness has been verified in a voltage range from 20 to 600 V, indicating its practicality in a broad nanoengineering applications.

## Additional file

Additional file 1: Figure S1. Linear correlation between AAO pore depth and integrated charge density in a large voltage range (a) 20–200 V; (b) 346–600 V.
