# Supplementary material for: A Highly Controllable Electrochemical Anodization Process to Fabricate Porous Anodic Aluminum Oxide Membranes
Source: Nanoscale Res Lett. 2015 Dec 26;10:495. doi: 10.1186/s11671-015-1202-y (PMC4691247; doi:10.1186/s11671-015-1202-y)
Supplement: Additional file 1: Figure S1. — Linear correlation between AAO pore depth and integrated charge density in a large voltage range (a) 20–200 V; (b) 346–600 V. [file 11671_2015_1202_MOESM1_ESM.pdf]

A Highly Controllable Electrochemical Anodization Process to Fabricate Porous Anodic Aluminum Oxide Membranes

Yuanjing Lin[1], Qingfeng Lin[1], Xue Liu[1], Yuan Gao[1], Jin He[2], Wenli Wang[3,4]*, Zhiyong Fan[1]*

[1]Department of Electronic and Computer Engineering, The Hong Kong University of Science and Technology, Clear Water Bay, Kowloon, Hong Kong, China SAR

[2]Shenzhen SOC Key Laboratory, Peking University-HKUST Shenzhen-Hong Kong Institution, Shenzhen, 518051, China

[3]College of Textile and Clothing Engineering, Soochow University, Suzhou, 215021, China

[4]National Engineering Laboratory for Modern Silk, Suzhou, 215123, China

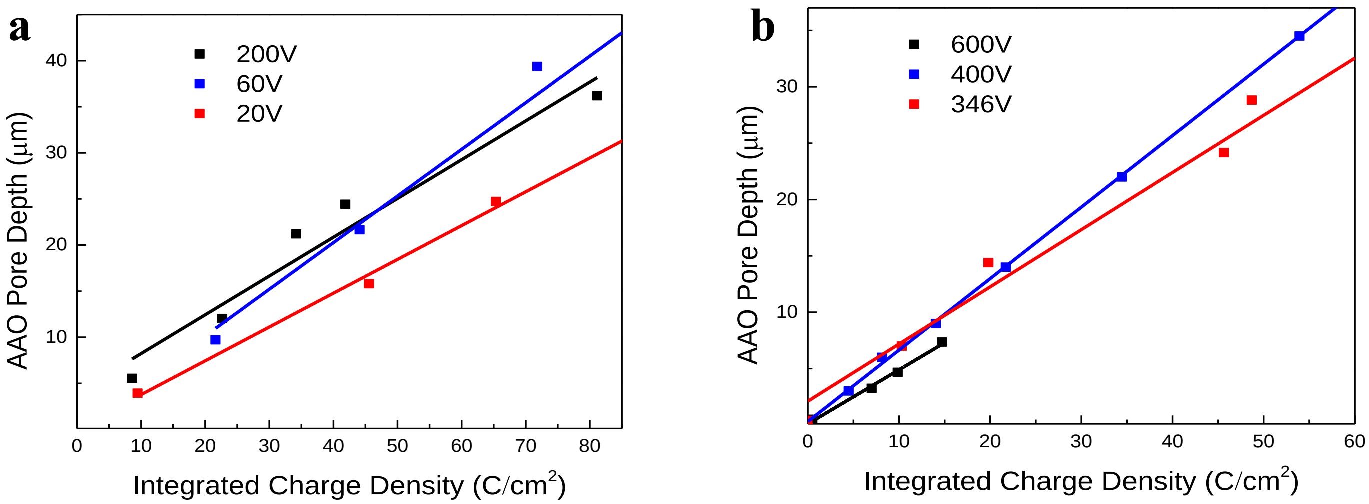

Figure S1. Linear correlation between AAO pore depth and integrated charge density in a large voltage range (a)20-200 V; (b) 346V-600 V.
